# Non-Hodgkin's lymphoma masquerading as splenic abscess: A case report

**DOI:** 10.1016/j.amsu.2022.104449

**Published:** 2022-08-18

**Authors:** Qaisar Ali Khan, Arif Mumtaz, Abdul Baqi, Hoor Ul Ain, Rabia Salman Mahfooz, Nowshad Asim, Sumaira Iram, Khabab Abbasher Hussien Mohamed Ahmed, Muhammad Junaid Tahir, Zohaib Yousaf

**Affiliations:** a Khyber Medical University, KMU-IMS, Kohat, Pakistan; b Mercy Saint Vincent Medical Center, Toledo, OH, United States; c Jinnah Medical College, Peshawar, Khyber Pakhtunkhwa, Pakistan; d Shalamar Institute of Health Sciences, Lahore, Pakistan; e Sultan Qaboos University and Hospital, Oman; f Faculty of Medicine, University of Khartoum, Sudan; g Lahore General Hospital, Lahore, 54000, Pakistan; h Tower Health, Reading, PA, USA

**Keywords:** Lymphocytes, Extranodal, Spleen, Computed tomography, Chemotherapy, Malignancy

## Abstract

**Background:**

Non-Hodgkin's lymphoma (NHL) is a monoclonal proliferation of lymphoid cells from B lineage. Although NHLs are primarily hematological tumors of lymph nodes but rarely can involve extranodal sites such as the spleen.

**Case report:**

A 29-year-old female patient presented with low-grade fever, cough, anemia, weight loss, tender left hypochondrium, and splenomegaly. A hypodense lesion in the spleen with central necrosis, having strong positivity for common leukocyte antigen (LCA), CD 20, and CD 10, led to a diffuse large B cell lymphoma diagnosis. The patient had an excellent clinical post-splenectomy response to combination chemotherapy and immunotherapy.

**Conclusion:**

NHL can present with symptomatic extra-nodal involvement without enlarged lymph nodes.

## Highlights

• Non-Hodgkin's lymphoma (NHL) is a group of malignant lymphoproliferative disorders (LPD) that primarily involve the lymph nodes.
• NHL is characterized by an irregular proliferation of T or B lymphocytes. Most NHLs are of B-cell origin.
• NHLs range from indolent malignancies (low-grade histology) to rapidly growing, highly aggressive tumors (high-grade histology).
• Extra-nodal involvement is common in the spleen, with prevalence being 20%–30%.
• Here we described a 29-year-old female patient presented with low-grade fever, cough, anemia, weight loss, tender left hypochondrium, and splenomegaly.

## 1 Introduction

Non-Hodgkin's lymphoma (NHL) is a group of malignant lymphoproliferative disorders (LPD) that primarily involve the lymph nodes [1]. NHL is characterized by an irregular proliferation of T or B lymphocytes. Most NHLs are of B-cell origin [2]. NHLs range from indolent malignancies (low-grade histology) to rapidly growing, highly aggressive tumors (high-grade histology) [3]. Extra-nodal involvement is common in the spleen, with prevalence being 20%–30%.^3^ NHLs that arise in the spleen are mostly limited to the spleen and its sentinel lymph nodes but can sometimes spread to other parts of the abdomen. Peripheral lymphadenopathy may or may not exist [4]. Splenomegaly is an atypical presenting feature of NHL. It is not usually the presenting symptom in NHL, but it might occur later [5]. The work has been reported in line with SCARE criteria [6].

Splenectomy may be necessary for diagnosing, staging, and managing splenic NHL. However, the incidence of splenectomy has reduced with the advent of modernized diagnostic radiographic evaluations and molecular analysis [1]. We present an atypical case of NHL characterized by splenomegaly with a central necrotic lesion, absence of lymphadenopathy, and a normal bone marrow analysis.

## 2 Case report

A 29-year-old previously healthy Pakistani female presented with fever, cough, and subjective weight loss for three months. The fever was gradual in onset, low grade, and was associated with rigors, chills, and night sweats. The cough was productive in nature with whitish sputum and was not associated with chest pain. A review of systems was remarkable for lethargy, malaise, and myalgias, for the past year. The patient never smoked and had no sick contact or a recent travel history.

On examination, the patient was alert and oriented. She had conjunctival pallor with no palpable lymph nodes. On presentation, the patient was afebrile (98 °F), blood pressure was 122/81 mmHg, pulse rate was 90 beats per minute, and respiratory rate was 18 breaths per minute. Abdominal examination revealed tender left hypochondrium along with splenomegaly. The rest of the examination was unremarkable.

A working diagnosis of likely pulmonary tuberculosis was made, and the patient was isolated with an airborne precautions protocol. Three sets of sputum analysis were negative for acid-fast bacilli (AFB) smear. The chest x-ray was unremarkable. The initial workup included a complete blood count (CBC), erythrocyte sedimentation rate (ESR), liver function test, renal function test, and three sets of blood culture. Concurrently, an echocardiogram was also done, considering the possibility of subacute endocarditis, but was unremarkable.

The ESR was significantly elevated (135mm/hr.), and the patient had severe microcytic hypochromic anemia with thrombocytopenia (Table 1). Transthoracic echocardiography showed no vegetations or valvular leaks.

**Table 1:** Investigations.

Investigation	value	Investigation	value
ESR White blood cells	135 mm/hr. 10.88 cells/μl	MCH	20 pg
Red blood cells	3.60 cells/μl	MCHC	29.3 g/dl
Hemoglobin	6.9 g/dl	Platelets count	140 10^3/μl
Hematocrit Serum Protein	24.6% 7.1 g/dl	Mean corpuscular volume	68.3 fl
Brucella titer	Negative	Malarial parasite	Negative

ESR: erythrocyte sedimentation rate; MCH: mean corpuscular hemoglobin; MCHC: mean corpuscular hemoglobin concentration; mm/hr:millimeter per hour; ul: microliter; g/dl:gram per deciliter; fl: femtoliter.

**Fig. 1:**
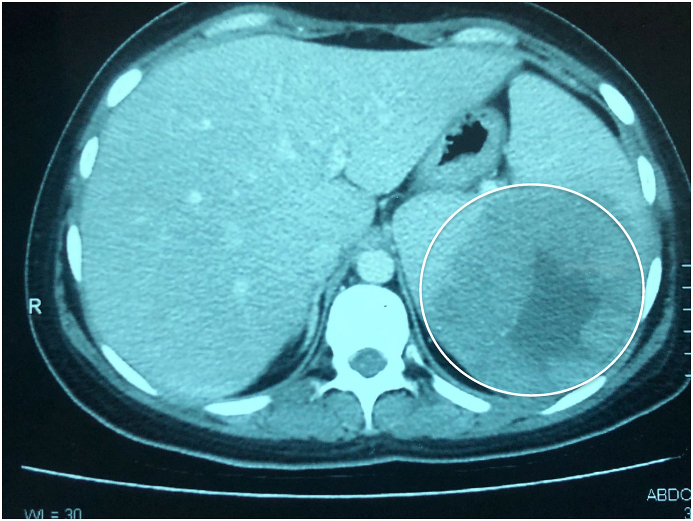
Computed tomography showed a homogenous 7 × 8cm hypodense splenic lesion with smooth borders and internal necrotic area—no evidence of enlarged abdominal lymph nodes.

Ultrasound of the abdomen showed an enlarged (15cm) spleen with a large hypoechoic lesion involving the spleen's upper and mid pole region measuring 8 × 9cm. A contrast-enhanced abdominal computed tomography scan was ordered and revealed a hypodense lesion in the spleen with central necrosis (Fig. 1).

A peripheral blood smear was unremarkable, and a bone marrow biopsy was done as part of the workup for cytopenia. The marrow was normocellular with active erythropoiesis, myelopoiesis, lymphopoiesis, and plasma cells.

A working diagnosis of non-Hodgkin's lymphoma (NHL) and a splenic abscess was made. She was treated with broad-spectrum antibiotics with no improvement. Three pints of packed red cell concentrate were transfused. Due to a lack of local expertise on the percutaneous aspiration of the splenic abscess, the patient was vaccinated against Pneumococcus, Meningococcus, and Haemophilus Influenzae, followed by a splenectomy.

Histopathology report of the splenic specimen showed a diffuse large B cell lymphoma with strong positivity for common leukocyte antigen (LCA), CD-20, and CD-10 with negativity to pan-keratin, CD-30, and CD-5, which favored a diagnosis of diffuse large B cell (DLBL) subtype.

The patient was started on a cyclical combination of chemo-immunotherapy, including cyclophosphamide, hydroxydaunorubicin, rituximab prednisolone, and vincristine. The patient improved clinically within three months of treatment and is now in remission.

## 3 Discussion

NHL is a common hematologic malignancy of the spleen [7]. NHL can present with splenomegaly or a well-defined hypoechoic mass on imaging. Anechoic regions in the spleen on imaging can be challenging to distinguish from a splenic abscess, especially if a patient is febrile.

Our patient was anemic, having chronic low-grade fever, weight loss, and splenomegaly. The initial laboratory evaluation showed increased ESR. Abdominal ultrasound showed a massive hypoechoic lesion in the spleen's upper and mid-pole regions and a hypodense lesion in the spleen with central necrosis without enlarged/reactive lymph nodes on a computed tomographic (CT) scan, leading to the suspicion of a splenic abscess.

Most physicians agreed to treat it as a splenic abscess, and the patient was started on broad-spectrum antibiotics, but the patient did not respond to antibiotics. Due to a lack of percutaneous aspiration facility, the patient underwent a splenectomy. The sample was sent for histopathological and immunohistochemical analysis evaluation, which led to the diagnosis of a high-grade diffuse large B cell lymphoma (DLBL).

NHL infiltrates the spleen in 40% of cases [5]. Systemic chemotherapy regimen R–CHOP is the first-line treatment [7]. However, most splenic NHL are treated with splenectomy as splenectomy is associated with low morbidity and mortality and can prevent hematological anomalies [5]. Splenectomy may prolong the initial disease treatment time but is associated with a 77% survival rate over five years [5]. Splenectomy reduces the chances of local recurrence and prevents metastasis to the other sites. Post-splenectomy-chemotherapy lessens recurrence and restricts neoplastic spread to other areas when used in conjunction with radiotherapy [8].

Splenic irradiation can be used if resection is not possible [9]. Post-splenectomy patients are more prone to get infected by a wide range of encapsulated microorganisms. Immunization regimens including Pneumococcal, Meningococcal, and H. Influenza vaccines must be given before splenectomy. Daily amoxicillin is given initially to prevent infections and can be used lifelong in an immuno-compromised state. Several cases of splenic lymphoma had previously been documented in the literature [3,4,[9], [10], [11]].

Our case was unique as there was only a solitary necrotic splenic lesion without enlarged reactive lymph nodes in high grade nhl which are typically an early sign of low grade nhl, sometimes lymph nodes may occasionally repeatedly swell and shrink over time. A late diagnosis of NHL can usually be confirmed by bone marrow (BM) biopsy, as most NHLs go on to involve the BM.[12]

## 4 Conclusion

Splenic NHLs are hematological neoplasms diagnosed through a biopsy and immunohistochemical analysis. Therefore, it is concluded that splenomegaly with chronic B symptoms without enlarged reactive lymph nodes can mislead the correct diagnosis of NHL. Histopathologic and immunohistochemical analysis is always needed for confirmation of NHL associated with splenomegaly. Careful evaluation and proper investigation in cases of splenomegaly are required for effective management and a better prognosis.

## Ethical approval

Not required.

## Sources of funding

No funds were granted for this work.

## Author contribution

A.M, Q.A.K, and A.B conceived the idea, A.M, Q.A.K, and N.A were responsible for data collection and acquisition of data. Q.A.K, A.M, H.U.A, N.A, A.B, S.I, M.J.T, and Z.Y performed the literature review and wrote the manuscript. Z.Y, R.S.M, K.A.H and M.J.T reviewed and critically revised the manuscript. All authors have approved the final manuscript.

## Trial registry number

1.Name of the registry: Not required.

2.Unique Identifying number or registration ID: Not applicable.

3.Hyperlink to your specific registration (must be publicly accessible and will be checked):

## Guarantor

The Guarantor is the one or more people who accept full responsibility for the work and/or the conduct of the study, had access to the data, and controlled the decision to publish.

## Consent

Written informed consent was obtained from the patient to publish this case report and any accompanying images.

## Provenance and peer review

Externally peer reviewed, not commissioned.

## Declaration of competing interest

No conflicts of interest to declare
